# Omics-based molecular techniques in oral pathology centred cancer: prospect and challenges in Africa

**DOI:** 10.1186/s12935-017-0432-8

**Published:** 2017-06-05

**Authors:** Henry A. Adeola, Olujide O. Soyele, Anthonio O. Adefuye, Sikiru A. Jimoh, Azeez Butali

**Affiliations:** 1 Department of Oral and Maxillofacial Pathology, Faculty of Dentistry, University of the Western Cape and Tygerberg Hospital, Cape Town, South Africa; 2 International Centre for Genetic Engineering and Biotechnology, Cape Town, South Africa; 3 Division of Dermatology, Department of Medicine, Faculty of Health Sciences and Groote Schuur Hospital, University of Cape Town, Cape Town, South Africa; 4 Department of Oral Maxillo-facial Surgery and Oral Pathology, Obafemi Awolowo University, Ile-Ife, Nigeria; 5 Division of Health Sciences Education, Faculty of Health Sciences, University of the Free State, Bloemfontein, South Africa; 6 Department of Anatomical Sciences, Faculty of Health Sciences, Walter Sisulu University, Mthatha, Eastern Cape South Africa; 7 Department of Oral Pathology, Radiology and Medicine, University of Iowa, Iowa City, IA USA

**Keywords:** Omics-based, Molecular, Developing world, Oral pathology, Challenges

## Abstract

**Background:**

The completion of the human genome project and the accomplished milestones in the human proteome project; as well as the progress made so far in computational bioinformatics and “big data” processing have contributed immensely to individualized/personalized medicine in the developed world.

**Main body:**

At the dawn of precision medicine, various omics-based therapies and bioengineering can now be applied accurately for the diagnosis, prognosis, treatment, and risk stratification of cancer in a manner that was hitherto not thought possible. The widespread introduction of genomics and other omics-based approaches into the postgraduate training curriculum of diverse medical and dental specialties, including pathology has improved the proficiency of practitioners in the use of novel molecular signatures in patient management. In addition, intricate details about disease disparity among different human populations are beginning to emerge. This would facilitate the use of tailor-made novel theranostic methods based on emerging molecular evidences.

**Conclusion:**

In this review, we examined the challenges and prospects of using currently available omics-based technologies *vis*-*à*-*vis* oral pathology as well as prompt cancer diagnosis and treatment in a resource limited setting.

## Background

As the field of oral pathology expands in Africa, currently emerging omics-based molecular techniques are, in principle, poised to improve the oral disease diagnosis and treatment [1–4]. Despite the vast ground covered by the advances in molecular and omics-based technologies in the developed world, there remains a gap in the uptake and application of these methods in developing countries due to existing militating factors. In order for Africa not be left behind in all these highly beneficial technologies, innovation and maximization of the existing infrastructure is highly required. A sine qua non to research and innovative discoveries is good record keeping; which remains sub-optimally practiced in most developing economies [5–9]. For example, it has been historically recorded that two previous presidents of the United States of America (Ulysses Simpson Grant & Stephen Grover Cleveland) were diagnosed with oral cancer [10, 11]; however such information is lacking on how many African presidents have had oral cancer in the past. In fact, history has it that some celebrities like Sigmund Freud, the father of modern psycho-analysis; Giacomo Puccini, a famous opera composer; and Sammy Davis Jr., a leading entertainer, died of head and neck cancer [11].

Accurate capture of disease burden in Africa would provide impetus for addressing prevalent early diagnosis and treatment monitoring bottlenecks. The emergence of high throughput omics based sciences in the post genomic era concomitantly attracts the application of computational biology and bioinformatics to elucidate various omics based data [4, 12–14]. Most challenges preventing the implementation of omics-based molecular approaches in routine diagnostic oral pathology in Africa are human resources and infrastructure. Low educational levels; lack of disease registries; poor funding and fiscal policies; lack of biospecimen repositories; and political unrests, inter alia; have significantly impeded research activities in many countries in Africa [15, 16].

This paper focuses on emerging omics-based techniques and their diagnostic and therapeutic potentials and challenges in the context of a resource limited setting.

## Main text

### Historical perspectives and general diagnostic challenges

Basic biomedical laboratory sciences provides the scientific foundation of clinical practice and supports the use of novel scientific discoveries to justify clinical decision making [17]. However, there remains widespread disconnect between clinicians and basic medical scientists [18–21]. Granted the importance of the art of medicine and clinical practice [22], there is a significant necessity to embrace evidence based science in the era of precision medicine. This point was alluded to over a century ago by the Flexner Report of 1910 [23], which was employed to transform the medical education model in America by establishing integrated biomedical training systems as the gold standard. A shortage of needed infrastructure and manpower has fixated a significant proportion of medical research efforts in Africa on bedside practice; albeit medical practice from ancient Egyptian papyri has been documented for various aliment as early as around 2000 B.C [24–28]. Indeed, evidence of various primary and metastatic cancers has been found by paleopathological and archeological examination of Egyptian mummies [29–34]. Despite the antiquity of medical practice in Africa relative to other regions of the world, there still exists a paucity of application of novel omics-based approaches to routine diagnostic medical sciences.

Inequalities in social determinants of health in low and middle income countries such as those in sub-Saharan Africa constitutes a huge challenge to health care access [35–42]. In addition, there are ample evidences of the existence of ethnic-based disparities in health risks profile in many countries [43–47]. The prospect of using omics-based techniques under such daunting conditions in resource-limited settings is dismal. To set up the right atmosphere for routine diagnostic and therapeutic application of these merging omics-based techniques, systems have to be instituted to address these prevalent disparities in healthcare practice and access in sub-Saharan Africa.

Globally, these are interesting times to apply omics technologies in order to improve human health. Unfortunately, the African continent is way behind in terms of the financial and institutional commitment required to successfully implement a genomics program for research and clinical use. Genomics is a multi-million dollar endeavor with far reaching implications for a healthy and productive continent [48]. It will unravel health risk, accelerate drug discoveries and motivate lifestyles [49, 50]. Of the sub-Sahara African nations, only South Africa is investing in genomics technologies despite the success stories reported in developed countries around the world. For instance, Nigeria is a country of about 200 million people and there is no genome center despite the training and collaborative opportunities presented through the Human Heredity and Health in Africa (H3Africa) Initiative (h3africa.org/) [51], which facilitates genomic researches and manpower development across Africa. Other factors that discourage the application of molecular research and emerging omics-based techniques to routine diagnostic oral pathology practice in Africa includes: poor access to research journals and conferences [52]; lack of needed skilled manpower [53, 54]; oral pathologist-to-population ratio (including workload and interest) [53, 55]; lack of well-equipped infrastructure such as laboratories and clinics [56–58]; poor internet facilities [59]; unstable electricity/power supply [60]; unfavorable health policies [61–63]; poor collaborative team science [64, 65]; knowledge gaps/educational levels [66–68]; war/local unrest [69]; lack of disease registries [70]; data/record gap in hospitals units [70, 71]; and religious/cultural beliefs [72], inter alia.

### Molecular diagnostic challenges

The mortality rate of oral cancer is extremely challenging and depends mainly on the staging of disease at diagnosis and commencement of treatment. Even, though the 5-year survival rate for first stage oral cancer cases can be as high as 80%, the 5-year survival rate for advanced stages (III/IV) are dismally low (20%); Up to 50% of oral cancer cases globally are only detected in late stages [73]. Hence the use of emerging diagnostic approaches to improve early diagnosis and prompt commencement of treatment is key in reducing the high mortality of oral cancer.

**Fig. 1:**
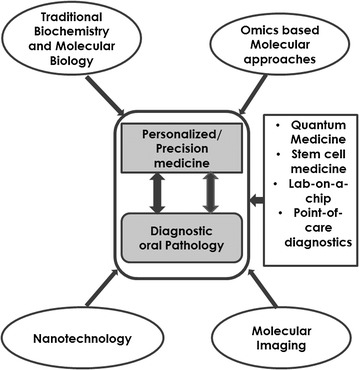
Impact of omics based molecular approaches on personalized/precision medicine and the field of diagnostic oral pathology

A fundamental goal of surgical pathology is to distinguish benign lesions from malignant ones [74, 75]. It is also equally important to be able to differentiate between indolent and aggressive tumors [76]. The use of hematoxylin and eosin (H&E) staining as well as the use of various special stains; backed with good clinicopathologic acumen, has partly improved the diagnosis of disease, albeit this is sometimes with limited diagnostic accuracy [77, 78]. The introduction of immunohistochemistry into diagnostic pathology significantly improved the confirmation of diagnoses, where morphological differential diagnosis using H&E presented a dilemma. However, immunohistochemistry has been cautiously used and interpreted after its limitations (such as variable antibody reactivity, background staining, poor quantitation, and subjective interpretation) became apparent [78–85]. Another layer of complexity is added to the diagnostic dilemma by intratumour heterogeneity and inter-biopsy heterogeneity, which presupposes that multiple cancer molecular signals can be detected in various sampled regions [86, 87]. This alludes to the notion that molecular classification of disease may be complimentary and in some situations more important than conventional histopathological diagnosis based on H&E staining [88–92]. The advent of various omics based molecular approaches as described hereafter, can potentially improve the diagnosis and monitoring of disease, particularly in the diagnostic grey areas. The benefit of using multiple high throughput techniques in a complementary and integrated manner would no doubt benefit personalized/precision medicine and the field of diagnostic oral pathology immensely (Fig. 1).

### Current and future molecular approaches

The field of molecular biology has undergone significant evolution in the post-genomic era [93–97]. With the emergence of omics-based approaches, research capabilities have expanded from low to medium throughput biochemistry, to interrogation of the full complement of biomolecules in a high throughput manner. Further, biological molecule have now been characterized in a manner that was hitherto not possible [97]. A few relevant high throughput omics based methods are described hereafter as they relate to the field of oral pathology and cancer.

#### Traditional biochemistry/molecular biology

To improve the field of molecular medicine, traditional biochemistry has employed various approaches such as: electrophoresis; Western, Northern, and Southern—blotting techniques for protein, RNA and DNA respectively [98]; enzyme-linked immunosorbent assay (ELISA) [99]; gene silencing and RNA interference [100]; gene cloning [101]; conventional and real-time qualitative polymerase chain reaction (PCR) [102]; karyotyping & fluorescence in situ hybridization (FISH) [103]; Comparative genomic hybridization (CGH) [104]; and chromosomal/cytogenetic analysis [105]. However, many of these techniques are limited because they are low to medium throughput in their capabilities. These techniques have benefitted the field of oral pathology by enabling the identification of molecular markers of various diseases. For example, cytogenetic alterations such as copy number gain of 16q, 8q and loss 3p, 8p, 9p, 4q, 5q, 13q have been found to be biomarkers for premalignant oral lesions; while copy number gain of 3q, 8q, 9q, 20q, 7p, 11q13, 5p and copy number loss of 3p, 9q, 21q, 5q, 13q, 18q, 8p have been found to characterize oral squamous cell carcinoma [106–109]. Molecular alterations such as microsatellite instability (MSI), abnormal mismatch repair protein (MMR) proteins MLH1, PMS2, MSH2, MSH6, and loss of heterozygosity (LOH) of 9p21, 3p14 have been found to characterize premalignant oral lesions [106–108, 110, 111]; while perturbation of *p53, EGFR/STAT, COX*-*2, NF*-*κB, VEGF, TGF*-*β/Ras* pathways have been found in oral squamous cell carcinoma [112–114]. Identified potential biomarkers of metastatic oral squamous cell carcinoma includes E-cadherin, integrins, matrix metalloproteinases (*MMP*s), IL-8, chemokine receptor 7 and *EGFR* [111]. Various fusion oncogenes have been used as potential biomarkers of salivary gland tumours; such as *MYB*-*NF1B* t(6:9)(q22-23:p23-24) for Adenoid cystic carcinoma [115]; *CRTC1*-*MAML2* t(11:19)(q21-22:p13) for low or intermediate grade Mucoepidermoid carcinoma [116]; *ETV6*-*NTRK3* for Mammary analogue secretory Carcinoma [116, 117]; *PLAG* & *HMGA2* for Pleomorphic adenoma [118, 119]; *EWSR1*-*POU5F1* t(6:22)(p21:q12) for high grade Mucopeidermoid carcinoma [116]; *EWSR1*-*ATF1* t(12:22)(q15:q12) for low grade hyalinizing clear cell carcinoma [120]; *NUT*-*BRD4* t(15:19)(q14:p13.1) for NUT midline carcinoma [121, 122]; and *MECT1*-*MAML2* for low grade Mucoepidermoid carcinoma [123]. Considering the impact of tradition molecular biology advances on diagnosis of tumors in the head and neck region, it is plausible that emerging high throughput omics based techniques would even bring greater breakthroughs to diagnostic oral pathology practice.

#### Omics based approaches

Prior to the completion of the human genome project (which costed billions of dollars and lasted over a decade), only short fragments of DNA could be sequenced using methods such as polymerase chain reaction and hybrid capture [124, 125]. However, with the advent of massive parallel sequencing (also known as Next Generation Sequencing), millions of DNA fragments can now be sequenced even without prior knowledge of the sequence [124]. With an exponential reduction in the cost of sequencing, Next Generation Sequencing (NGS) has improved the utility of various omics field in understanding disease specific genomes [125].

The field of genomics and sequencing also owes its huge success to the development of the array technologies, which were initially fabricated for high throughput genomic interrogate the transcriptional levels of thousands of genes in a single experiment [126, 127]. This technology has made it possible to evaluate pathophysiological gene expression patterns in cells and tissues; as well as to identify drug targets in tissues [126]. Different types of arrays and their application for various biological functions have been discussed in details elsewhere [126–128].

In addition, emerging technological advances have provided the unique opportunity to interrogate biological and genomic complexity to the single-cell resolution. This potentially provides high throughput omics based data which helps to delineate tissue heterogeneity from a bulk population of cells; as well as diversity in complex microbial ecosystems [129]. Although technically challenging, single cell technology has been applied both to genomic and epigenomic analyses of diseases [129, 130]; as well as to drug discovery and development [131].

In tandem with the ever-increasing amount of data generated from high throughput data, a great number of omics fields have emerged [132]. These omics fields have provided access to systems level interpretation of molecular processes. However these techniques requires robust bioinformatics and computational infrastructure to de-convolute and integrate the emerging data for clinical utility [14]. The -omics suffix indicate the analysis of the full complement of a specific biomolecule; as well as its characterization, interaction or analysis [133]. For example, the measurement of the full complement of protein in a cell, tissue, body fluid, or any biological system is known as proteomics; and this analogy applied to all other biomolecules such as lipids (lipidomics), genes (genomics), gene transcripts (transcriptomics), metabolites (metabolomics), etc. It has been notably demonstrated by Garcia et al. [134], that such omics based techniques would benefit personalized oral healthcare immensely. Examples of promising application of different omics based approaches are described below:

##### Genomics

Genomics techniques provide a genome-wide access to genetic information and presents a robust opportunity to interrogate cancer biology in a high throughput manner. Genomics information have been used to develop databases that have enhanced our knowledge of the cancer genome expression greatly [135]. Although, genomics has attained moderate success in target oncogene and tumor suppressor gene identification; there remains significant challenges in the transformation of these targets into therapies that would improve cancer patient management [136]. Application of genomics to oral cancer diagnosis in diagnostic oral pathology would be greatly improved by advances in the field of dental and craniofacial informatics [137]. Genomic alterations have been identified for leukoplakia as well as in the process of sequential oral tumorigenesis [138]; providing molecular information that were hitherto unavailable.

##### Transcriptomics

Differential transcriptomics profiling of oropharyngeal cancers based on human papilloma virus (HPV) status has been shown to provide reliable molecular signature to stratify these subtypes of head and neck cancer [139]. Thus permitting high throughput analyses of gene transcript and drawing of biological inferences on HPV-related oral cancer.

##### Genome-wide association studies (GWAS)

Genome-wide association studies (GWAS) is an unbiased statistical approach used to identify common single nucleotide polymorphisms across the genome that are associated with complex traits. Since the early 2000s when it was first used, there have been over 2000 published GWAS studies [140]. The success of GWAS is largely dependent on the coverage of the genotyping panel, the minor allele frequency of SNPs in the investigated population and on clearly defined phenotypes. GWAS has been used to identify many novel susceptibility loci for complex traits including oral cancers [141].

##### Next generation sequencing (NGS)

Deep sequencing, massively paralleled sequencing or next generation sequencing (NGS) is a novel DNA or RNA sequencing technology that has transformed genomic research [142]. As opposed to the Sanger sequencing, this is a high throughput method that can be used to sequence the complete human genome within a day [143]. Significant progress in the sequencing research has led to a reduction in its per megabase cost, number of produced sequence reads per run as well as the genome diversity coverage; which helps to adequately elucidate complex phenotypes of diseases [142, 144]. NGS has been applied to understand oncogenic mutations in oral diseases such as ameloblastomas [145, 146]. Using this method, it was discovered that mutations in the *SMO* gene encoding smoothened protein was commoner in maxillary ameloblastomas, while *BRAF V600E* mutations were commoner in mandibular ameloblastomas [145]. This has far reaching implications for the application of personalized medicine to the management of ameloblastomas [146]. Molecular heterogeneity in head and neck cancers has also been elucidated using NGS methods [147].

##### Whole exome sequencing (WES)

There is an increasing confidence in our ability to understand the impact of identified coding variations. In addition, we are able to sequencing the entire protein coding regions in the genome also known as exome sequencing. Therefore, exome sequencing appears to be a promising omics tool for the rapid identification of functional variations. These thus provide an opportunity for small molecule development through pharmacogenomics and also serve as information for counselling to at-risk families with diseases. In recent times, exome sequencing was used to identify novel oral cancer genes and loci [148–151].

##### Epigenomics

The study of stable and often heritable changes in gene expression patterns that are not caused by changes in the DNA sequence is known as epigenetics [152]. Two of the most well characterized epigenetic alterations are histone modification and DNA methylation [153]. Beyond the genome, the complete set of epigenetic modifications to the cellular DNA or histones (epigenome) are known to play an important role in the etiology of diseases [154]. The epigenome plays a pivotal role in the regulation of chromatin activity and therefore affect DNA repair and gene expression [152]. Epigenomic alterations have been established in obesity, diabetes and cancer [154–156]. Adequate evidence exists, that epigenetic dysregulations have been implicated in the pathogenesis of oral and oropharyngeal cancers [157–161]; hence it is plausible that epigenomic analyses would provide better insight into oral carcinogenesis.

##### Microbiomics

Bacterial genetics of the human oral microbiota has been interrogated using a combination of transcriptomics and microbiomics techniques [162–164]. This techniques is highly beneficial for understanding infective dental pathologies such as periodontitis, osteomyelitis and caries; and can be potentially applied to non-infective diseases such as cancer as well [165, 166].

##### Proteomics

Mass spectrometry-based quantitative proteomics analysis has revealed an enhanced interferon-related signaling pathway for oral cancer cells in vitro, using labeled-mass spectrometry coupled to a high performance liquid chromatography (HPLC) system [167]. Such findings required further study into the significance of interferon in the pathogenesis of oral squamous cell carcinoma and may serve as a basis for development of targeted therapies and potential biomarkers for oral cancer.

##### Lipidomics

One of the major breakthroughs in the field of lipidomics that could potentially revolutionize the field of surgical oral pathology is the fast, real-time mass spectrometry based identification of surgical margin of tissues intraoperatively with the use of the i-knife [168]. This technique diagnosed cancer margin accurately in a more reliable and unbiased manner using lipidomic signatures that differentiated between tumor and normal areas [168]. This could potentially eliminate the intraoperative waiting time while sending surgical specimen for frozen section tumor margin analysis.

##### Metabolomics

The science of metabolomics looks at the differential signature of metabolites in biological pathways in a high throughput manner. Tiziani et al. [169] identified metabolomics signatures for early diagnosis or oral cancers using ^1^H-nuclear magnetic resonance (NMR) spectroscopy methods. This method could potentially be used for routine early clinical diagnosis of various oral cancers. Recently, the push for reliable non-invasive timely diagnosis of cancer has directed research interest in the area of exhaled breath analysis for early detection. Breathomics is a branch of metabolomics that measures the total amount of volatile organic compounds (VOCs) in exhaled air [170]. Volatile and nonvolatile organic components of the exhaled air are relevant indicator of metabolic status for clinical diagnosis and monitoring purposes [171]. Various metabolic processes in the body produce VOCs that are released into the blood and transported to the lung where they are passed to the airway and exhaled. Acquisition and measurement of unique VOCs that may indicate occurrence of chronic inflammation and/or oxidative stress are potential biomarkers for early cancer detection [172]. This may be a plausible non-invasive early-stage cancer screening tool and may be potentially applied to the detection of head and neck cancers.

#### Ancillary tools for omics based technology

##### Nanotechnology

Nanotechnology is an emerging, highly beneficial, multidisciplinary area of research that deals with atomic and molecular levels of matter. Some clinical trials are currently directed at demonstrating the theranostic efficacy of nanomaterials against chronic diseases such as cancer [173]. Today, nanomedicine plays a significant role in diagnostic sciences, gene therapy, drug delivery systems, as well as in screening of populations [174, 175]. Both the field of medicine and dentistry have benefitted reasonably from therapeutic and diagnostic applications of nanomaterials [176]. Several forms of nanomaterials and nanotechnology methods have been used for the diagnosis and treatment of oral cancer. A few such modalities that have benefitted the field of oral pathology and oral cancer diagnosis and treatment are Surface Enhanced Raman Spectroscopy (SERS) [177, 178], composite organic–inorganic nanoparticles (COIN) [179, 180] and quantum dots (QD) [177, 181]. For example, Raman difference spectroscopy has been demonstrated as a non-invasive method for oral cancer diagnosis [182]. There is no doubt however; that these and many other nanotechnology approaches would continue to enhance the application omics approaches to personalized medicine and oral pathology.

##### Molecular imaging

Molecular imaging is a highly beneficial tool with the capacity to improve every aspects of cancer care. It is an in vivo imaging-based characterization and measurement of the key biomolecules and molecular events that are basic to the malignant or aberrant state [183]. Prior to the emergence of molecular imaging, a number of “gold standard” scientific approaches (such as ViziLite, VELscope, Trimira and OralCDx, etc.) aimed at oral lesion detection were fraught with inconsistencies during standard routine head and neck examinations [184–186]. However, the establishment of integrated MRI/PET has improved the consistency and effectiveness of earlier stage cancer detection [187]. Molecular imaging such as positron emission tomography (PET) often integrated with cross sectional imaging in the form of PET/computed tomography (PET/CT), PET/magnetic resonance imaging (MRI)/MR spectroscopic imaging (MRSI), as well as optical imaging; play a vital role in cancer detection, staging and assessment of treatment response. The optical imaging is mostly performed with the radiotracer ^18^F-fluoro-2-deoxy-d-glucose [FDG], integrated with cross-sectional imaging in the form of PET/computed tomography (PET/CT) [188]. PET has been said to be the leading molecular imaging approach in a clinical environment [189–191]. PET imaging methods have been successful in both staging of diverse cancers and assessment of response of tumors to therapy [192, 193]. Several authors have shown a significantly higher level of sialic acid in oral cancer patients when compared to normal patients [194–196]. The recent discovery of molecular imaging-based individualized potential molecular tumor fingerprint has facilitated a rapid and effective development of theranostic drugs for novel treatment algorithms [197, 198]. In another study, the efficacy of fluorescence imaging using topically applied lectin-fluorophore conjugates as compared to conventional tissue autofluorescence in distinguishing tumor from normal tissues was also investigated [199]. The results revealed that the changes in glycosylation could differentiate normal from cancerous tissues in the oral cavity with high SNRs [199]. This is potentially a non-invasive screening method for premalignant and malignant oral mucosal tumors; and as a method for defining surgical margins and monitoring cellular changes over time. To further validate this approach for oral cancer screening, in vivo testing in a larger clinical cohort is needed. Not least, Nanobodies have also been considered as highly beneficial agent in molecular imaging of cancers, due to its rapid accumulation in tumors, homogenous distribution; efficient blood clearance, high specificity, safety, high tumor signal-to-background ratios; as well as ease of conjugation to several kinds of imaging techniques [200].

##### Future molecular concepts

Several advances have emerged in precision and personalized medicine which could potentially benefit the field of oral pathology vis-à-vis molecular oral cancer diagnostics and therapy. The advent of microfluidic technology [201, 202] has made it possible to establish a rapid multistage, multi-technique technology known as Lab-on a-chip [202, 203]. This has permitted a high turnover of requested laboratory investigations during clinical diagnosis and therapy. This technology and those mentioned above have rapidly improved the development of point-of-care (POC) diagnostic tools [204–206]. These developments may have potential applications for oral pathology and cancer management. It is also clear that stem cell science has improved the field of dentistry and oral pathology. Somatic stem cells can be harvested from patients and reprogrammed to form patient-specific induced pluripotent stem (iPS) cells [207]. These iPS cells can be used for recombination and regenerative production of maxillofacial structures for transplantation and maxillofacial structure reconstruction [207]. On the other hand, subpopulations of cancer stems cells have been previously identified in head and neck cancers, by the application of stem cell science [208]. Such in-depth knowledge can also provide future stem cell-based targeted therapies against head and neck cancers [209]. Quantum medicine approaches such as quantum tunneling [210] has been previously used in understanding genetic mutations in cancers [211]. A quantum mechanical approach is now being considered in the understanding of the evolution of cancers [211, 212]. There is no doubt that these emerging molecular concepts are poised to play a major role in oral pathology and cancer diagnosis; as well as therapies in the foreseeable future.

### Recommendations

Considering the immense potential benefits of omics based approaches in the field of oral pathology and cancer diagnosis in developing African regions, the authors make the following recommendations:

- Government focus should be directed at funding Infrastructure (bridging the record gap); funding researchers and supporting research training (bridging the knowledge gap).
- As custodians of various tissue specimens, pathologists must take the lead (and must not be passive) in the application of omics based molecular techniques to routine diagnostic services. Advanced certification and annual remedial courses are also recommended.
- With favorable health policy change, omics based molecular approaches should be integrated into routine clinical practice, taking dutiful quality assurance (internal and external) measures.
- There should be private sector/non-governmental organization (NGO) participation to make the task of integration of omics into oral pathology effective.
- Reimbursement policy for oral pathologist who are willing to practice omics science must be favorable.
- Legislative initiative must be available to pass this concept into law.
- Scarce resources must be maximized (using mobile phones, internet, etc. to improve the practice of omics based approaches in oral pathology),
- Viable collaborative team science established (sharing ideas, research, equipment and meetings) must be established locally, regionally, continentally and globally.
- Research and Educational Networks (RENs) must be established using a trans/inter/multidisciplinary approach
- Omics-based science and personalized medicine topics should be integrated into the undergraduate and postgraduate medical/dental training curriculum
- There are many freely available online platforms that tremendously facilitate omics based techniques, such as the Gene Expression Ominbus (GEO) [213]; National Center for Biotechnology Information (NCBI) [214]; and The Cancer Genome Atlas (TCGA) [215]. Such platforms offer great opportunities to develop knowledge in the omics field and researchers should be well enlightened about this.

## Conclusion

In the light of the aforementioned recommendations and the tremendous burden of cancer in Africa, healthcare goals needs to capture the most reliable and cost effective methods for screening and early diagnosis of disease. It is unfortunate that despite the fact that up to 80% of the burden of cancer is found in the low and middle income countries (LMIC), it only receives about 5% of the global spending on cancer [216]. Africa has to piggy-back and emulate already existing transformative “training-the-trainer” systems in the Western world such as the: Training Residents in Genomics program (TRIG) and the Resident in Service Examination (RISE) practiced in the Americas and Western Europe [217, 218]. These programs exposes trainees to hands-on omics based molecular approaches during their residency program; and thus increases their confidence in requesting for and interpretation of such investigations. Considering that the cost of genomics investigation is on the decline and that we have entered into the $1000 genome era [219, 220], the pertinent question for African oral pathologists is “are you ready for a genome-related clinical visits (with respect to their genetic risk for oral pathologies) by patients?” It is plausible that future histopathological reports would proceed beyond classic histological findings to morpho-molecular findings [218]; and a good knowledge of omics based molecular techniques is a *sine qua non* for an astute diagnostician. Emphasis should be placed on the multimodality approaches for omics based diagnostic oral oncological practices. Although all these techniques improve our knowledge of disease biology in an in-depth manner, they are most likely to play an adjunctive/supportive role rather than replacing existing pathological techniques in its application for improving detection and prognostic evaluation of head and neck cancer.

## Abbreviations

B.C: before christ
CGH: comparative genomic hybridization
COIN: composite organic–inorganic nanoparticles
CT: computed tomography
DNA: deoxyribonucleic acid
ELISA: enzyme-linked immunosorbent assay
FDG: ^18^F-fluoro-2-deoxy-d-glucose
FISH: fluorescent in situ hybridization
GWAS: genome-wide association studies
H&E: hematoxylin and eosin
HPLC: high performance liquid chromatography
HPV: human papilloma virus
iPS: induced pluripotent stem
LMIC: low and middle income countries
MRI: magnetic resonance imaging
MRSI: magnetic resonance spectroscopic imaging
MSI: microsatellite instability
NGO: non-governmental organization
NGS: next generation imaging
NMR: nuclear magnetic resonance PET
POC: point-of-care
QD: quantum dot
REN: research and educational network
RISE: resident in-service examination
RNA: ribonucleic acid
SERS: surface enhanced Raman spectroscopy
SNR: signal-to-noise ratio
TRIG: training residents in genomics
VOC: volatile organic compounds

## References

[1] Glurich I, Acharya A, Brilliant MH, Shukla SK (2015). Progress in oral personalized medicine: contribution of ‘omics’. J Oral Microbiol.

[2] Tasoulas J, Patsouris E, Giaginis C, Theocharis S (2016). Salivaomics for oral diseases biomarkers detection. Expert Rev Mol Diagn.

[3] Koneru S, Tanikonda R (2014). Salivaomics—a promising future in early diagnosis of dental diseases. Dent Res J.

[4] Giacomelli L, Covani U (2010). Bioinformatics and data mining studies in oral genomics and proteomics: new trends and challenges. Open Dent J.

[5] Hollis AC, Ebbs SR (2016). An examination of inpatient medical record keeping in the Orthopaedic Department of Kilimanjaro Christian Medical Centre (KCMC), Moshi, Tanzania. Pan Afr Med J.

[6] Ross IL (2016). Exploring rare diseases in South Africa, a personal journey: time for electronic record-keeping. Ann Med Health Sci Res.

[7] Dosumu EB, Dosumu OO, Lawal FB (2012). Quality of records keeping by undergraduate dental students in ibadan, Nigeria. Ann Ib Postgrad Med.

[8] Teviu EA, Aikins M, Abdulai TI, Sackey S, Boni P, Afari E, Wurapa F (2012). Improving medical records filing in a municipal hospital in Ghana. Ghana Med J.

[9] Thomas J (2009). Medical records and issues in negligence. Indian J Urol.

[10] Renehan A, Lowry JC (1995). The oral tumours of two American presidents: what if they were alive today?. J R Soc Med.

[11] Folz BJ, Ferlito A, Weir N, Pratt LW, Rinaldo A, Werner JA (2007). A historical review of head and neck cancer in celebrities. J Laryngol Otol.

[12] Sung J, Wang Y, Chandrasekaran S, Witten DM, Price ND (2012). Molecular signatures from omics data: from chaos to consensus. Biotechnol J.

[13] Perco P, Rapberger R, Siehs C, Lukas A, Oberbauer R, Mayer G, Mayer B (2006). Transforming omics data into context: bioinformatics on genomics and proteomics raw data. Electrophoresis.

[14] Gomez-Cabrero D, Abugessaisa I, Maier D, Teschendorff A, Merkenschlager M, Gisel A, Ballestar E, Bongcam-Rudloff E, Conesa A, Tegner J (2014). Data integration in the era of omics: current and future challenges. BMC Syst Biol.

[15] Adewole I, Martin DN, Williams MJ, Adebamowo C, Bhatia K, Berling C, Casper C, Elshamy K, Elzawawy A, Lawlor RT (2014). Building capacity for sustainable research programmes for cancer in Africa. Nat Rev Clin Oncol.

[16] Gakunga R, Parkin DM, African Cancer Registry N (2015). Cancer registries in Africa 2014: a survey of operational features and uses in cancer control planning. Int J Cancer.

[17] Brass EP (2009). Basic biomedical sciences and the future of medical education: implications for internal medicine. J Gen Intern Med.

[18] National Research Council (US) Committee on the evaluation of the Lucille P. Markey charitable trust programs in Biomedical Sciences. Bridging the bed-bench gap: contributions of the Markey trust. A Bridge Building Between Medicine and Basic Science. Washington (DC): National Academies Press; 2004. https://www.ncbi.nlm.nih.gov/books/NBK215898/. 25009863

[19] Restifo LL, Phelan GR (2011). The cultural divide: exploring communication barriers between scientists and clinicians. Dis Model Mech.

[20] Arias IM (1989). Training basic scientists to bridge the gap between basic science and its application to human disease. N Engl J Med.

[21] Habbal OA, El-Mardi AM, Inuwa I (2007). The preclinical-clinical divide: building bridges. Sultan Qaboos Univ Med J.

[22] Leblond RF (2013). An epistemology for clinical medicine: an argument for reflection on the ends of medical practice and ways of knowing with implications for the selection and training of physician. Trans Am Clin Climatol Assoc.

[23] Duffy TP (2011). The Flexner report–100 years later. Yale J Biol Med.

[24] Stevens JM (1975). Gynaecology from ancient Egypt: the papyrus Kahun: a translation of the oldest treatise on gynaecology that has survived from the ancient world. Med J Aust.

[25] Saba MM, Ventura HO, Saleh M, Mehra MR (2006). Ancient Egyptian medicine and the concept of heart failure. J Card Fail.

[26] Karenberg A, Leitz C (2001). Headache in magical and medical papyri of ancient Egypt. Cephalalgia.

[27] Saber A (2010). Ancient Egyptian surgical heritage. J Invest Surg.

[28] Griffith FL (1893). A medical papyrus from Egypt. Br Med J.

[29] Binder M, Roberts C, Spencer N, Antoine D, Cartwright C (2014). On the antiquity of cancer: evidence for metastatic carcinoma in a young man from ancient Nubia (c. 1200 BC). PLoS ONE.

[30] Lieverse AR, Temple DH, Bazaliiskii VI (2014). Paleopathological description and diagnosis of metastatic carcinoma in an Early Bronze Age (4588 + 34 Cal. BP) forager from the Cis-Baikal region of Eastern Siberia. PLoS ONE.

[31] Prates C, Sousa S, Oliveira C, Ikram S (2011). Prostate metastatic bone cancer in an Egyptian Ptolemaic mummy, a proposed radiological diagnosis. Int J Paleopathol.

[32] Strouhal E (1978). Ancient Egyptian case of carcinoma. Bull NY Acad Med.

[33] Rehemtulla A (2010). Dinosaurs and ancient civilizations: reflections on the treatment of cancer. Neoplasia.

[34] Sudhakar A (2009). History of cancer, ancient and modern treatment methods. J Cancer Sci Ther.

[35] Bado AR, Appunni SS (2015). Decomposing Wealth-based inequalities in under-five mortality in West Africa. Iran J Public Health.

[36] Hajizadeh M, Sia D, Heymann SJ, Nandi A (2014). Socioeconomic inequalities in HIV/AIDS prevalence in sub-Saharan African countries: evidence from the Demographic Health Surveys. Int J Equity Health.

[37] Ataguba JE, Akazili J, McIntyre D (2011). Socioeconomic-related health inequality in South Africa: evidence from General Household Surveys. Int J Equity Health.

[38] Fox AM (2010). The social determinants of HIV serostatus in sub-Saharan Africa: an inverse relationship between poverty and HIV?. Public Health Rep.

[39] Eshetu EB, Woldesenbet SA (2011). Are there particular social determinants of health for the world’s poorest countries?. Afr Health Sci.

[40] Alam N, Hajizadeh M, Dumont A, Fournier P (2015). Inequalities in maternal health care utilization in sub-Saharan African countries: a multiyear and multi-country analysis. PLoS ONE.

[41] Worku EB, Woldesenbet SA (2015). Poverty and inequality—but of what—as social determinants of health in Africa?. Afr Health Sci.

[42] van Deurzen I, van Oorschot W, van Ingen E (2014). The link between inequality and population health in low and middle income countries: policy myth or social reality?. PLoS ONE.

[43] National Research Council (US) Panel on race, ethnicity, and health in later life. In: Anderson NB, Bulatao RA, Cohen B, editors. Critical perspectives on racial and ethnic differences in health in late life. Washington (DC): National Academies Press; 2004. doi:10.17226/11086. https://www.ncbi.nlm.nih.gov/books/NBK25532/. 20669464

[44] Egede LE (2006). Race, ethnicity, culture, and disparities in health care. J Gen Intern Med.

[45] Williams DR, Sternthal M (2010). Understanding racial-ethnic disparities in health: sociological contributions. J Health Soc Behav.

[46] Orach CG (2009). Health equity: challenges in low income countries. Afr Health Sci.

[47] Mays VM, Cochran SD, Barnes NW (2007). Race, race-based discrimination, and health outcomes among African Americans. Annu Rev Psychol.

[48] Hood L, Rowen L (2013). The human genome project: big science transforms biology and medicine. Genome Med.

[49] Urban MF (2015). Genomics in medicine: from promise to practice. S Afr Med J.

[50] Kumar D (2007). From evidence-based medicine to genomic medicine. Genomic Med.

[51] Butali A, Mossey P, Tiffin N, Adeyemo W, Eshete M, Mumena C, Audu R, Onwuamah C, Agbenorku P, Ogunlewe M (2015). Multidisciplinary approach to genomics research in Africa: the Africran model. Pan Afr Med J.

[52] Gray E (2013). Research for development and the role of ‘grey literature’ in southern African research production. Ecancermedicalscience.

[53] Naicker S, Plange-Rhule J, Tutt RC, Eastwood JB (2009). Shortage of healthcare workers in developing countries–Africa. Ethn Dis.

[54] Dodani S, LaPorte RE (2005). Brain drain from developing countries: how can brain drain be converted into wisdom gain?. J R Soc Med.

[55] Anyangwe SC, Mtonga C (2007). Inequities in the global health workforce: the greatest impediment to health in sub-Saharan Africa. Int J Environ Res Public Health.

[56] Essendi H, Johnson FA, Madise N, Matthews Z, Falkingham J, Bahaj AS, James P, Blunden L (2015). Infrastructural challenges to better health in maternity facilities in rural Kenya: community and healthworker perceptions. Reprod Health.

[57] Alemnji GA, Zeh C, Yao K, Fonjungo PN (2014). Strengthening national health laboratories in sub-Saharan Africa: a decade of remarkable progress. Trop Med Int Health.

[58] Stock R (1983). Distance and the utilization of health facilities in rural Nigeria. Soc Sci Med.

[59] Adedokun BO, Olopade CO, Olopade OI (2016). Building local capacity for genomics research in Africa: recommendations from analysis of publications in Sub-Saharan Africa from 2004 to 2013. Glob Health Action.

[60] Adair-Rohani H, Zukor K, Bonjour S, Wilburn S, Kuesel AC, Hebert R, Fletcher ER (2013). Limited electricity access in health facilities of sub-Saharan Africa: a systematic review of data on electricity access, sources, and reliability. Glob Health Sci Pract.

[61] Pfeiffer J, Johnson W, Fort M, Shakow A, Hagopian A, Gloyd S, Gimbel-Sherr K (2008). Strengthening health systems in poor countries: a code of conduct for nongovernmental organizations. Am J Public Health.

[62] Mooketsane KS, Phirinyane MB (2015). Health governance in sub-Saharan Africa. Glob Soc Policy.

[63] Rispel LC, de Sousa CADP, Molomo BG (2009). Can social inclusion policies reduce health inequalities in sub-Saharan Africa?—a rapid policy appraisal. J Health Popul Nutr.

[64] Whitworth JAG, Kokwaro G, Kinyanjui S, Snewin VA, Tanner M, Walport M, Sewankambo N (2008). Strengthening capacity for health research in Africa. Lancet.

[65] Chu KM, Jayaraman S, Kyamanywa P, Ntakiyiruta G (2014). Building research capacity in Africa: equity and global health collaborations. Plos Med.

[66] Valadez JJ, Berendes S, Jeffery C, Thomson J, Ben Othman H, Moxon S, Danon L, Turki AA, Saffialden R, Mirzoyan L (2013). Filling the knowledge gap: measuring HIV prevalence and risk factors among populations most vulnerable to HIV in Libya. Sex Transm Infect.

[67] Mufunda E, Wikby K, Bjorn A, Hjelm K (2012). Level and determinants of diabetes knowledge in patients with diabetes in Zimbabwe: a cross-sectional study. Pan Afr Med J.

[68] Jroundi I, Mahraoui C, Benmessaoud R, Moraleda C, Benjelloun B, Bassat Q (2015). Knowledge gaps on paediatric respiratory infections in Morocco, Northern Africa. Arch Public Health.

[69] Botha HP (1983). Primary health care according to African requirements. Isr J Med Sci.

[70] Jedy-Agba EE, Curado MP, Oga E, Samaila MO, Ezeome ER, Obiorah C, Erinomo OO, Ekanem IO, Uka C, Mayun A (2012). The role of hospital-based cancer registries in low and middle income countries—the Nigerian case study. Cancer Epidemiol.

[71] Mwaka AD, Wabinga HR, Mayanja-Kizza H (2013). Mind the gaps: a qualitative study of perceptions of healthcare professionals on challenges and proposed remedies for cervical cancer help-seeking in post conflict northern Uganda. BMC Fam Pract.

[72] Chukwuneke FN, Ezeonu CT, Onyire BN, Ezeonu PO (2012). Culture and biomedical care in Africa: the influence of culture on biomedical care in a traditional African society, Nigeria, West Africa. Niger J Med.

[73] van der Waal I (2013). Are we able to reduce the mortality and morbidity of oral cancer; some considerations. Med Oral Patol Oral.

[74] Tirumalae R, Roopa M (2013). Benign vs. Malignant skin adnexal neoplasms: how useful are silhouettes?. Indian J Dermatol.

[75] Leong AS, Zhuang Z (2011). The changing role of pathology in breast cancer diagnosis and treatment. Pathobiology.

[76] Hughes C, Murphy A, Martin C, Sheils O, O’Leary J (2005). Molecular pathology of prostate cancer. J Clin Pathol.

[77] Laine L, Lewin DN, Naritoku W, Cohen H (1997). Prospective comparison of H&E, Giemsa, and Genta stains for the diagnosis of *Helicobacter pylori*. Gastrointest Endosc.

[78] Fox H (2000). Is H&E morphology coming to an end?. J Clin Pathol.

[79] Matos LL, Trufelli DC, de Matos MG, da Silva Pinhal MA (2010). Immunohistochemistry as an important tool in biomarkers detection and clinical practice. Biomark Insights.

[80] Suster S, Moran CA (2006). Applications and limitations of immunohistochemistry in the diagnosis of malignant mesothelioma. Adv Anat Pathol.

[81] Preusser M, Wohrer A, Stary S, Hoftberger R, Streubel B, Hainfellner JA (2011). Value and limitations of immunohistochemistry and gene sequencing for detection of the IDH1-R132H mutation in diffuse glioma biopsy specimens. J Neuropathol Exp Neurol.

[82] Kadivar M, Boozari B (2013). Applications and limitations of immunohistochemical expression of “Napsin-A” in distinguishing lung adenocarcinoma from adenocarcinomas of other organs. Appl Immunohistochem Mol Morphol.

[83] Taylor CR, Levenson RM (2006). Quantification of immunohistochemistry–issues concerning methods, utility and semiquantitative assessment II. Histopathology.

[84] Dreux N, Marty M, Chibon F, Velasco V, Hostein I, Ranchere-Vince D, Terrier P, Coindre JM (2010). Value and limitation of immunohistochemical expression of HMGA2 in mesenchymal tumors: about a series of 1052 cases. Mod Pathol.

[85] Hofman F (2002). Immunohistochemistry. Curr Protoc Immunol.

[86] Yap TA, Gerlinger M, Futreal PA, Pusztai L, Swanton C (2012). Intratumor heterogeneity: seeing the wood for the trees. Sci Transl Med.

[87] Gerlinger M, Rowan AJ, Horswell S, Larkin J, Endesfelder D, Gronroos E, Martinez P, Matthews N, Stewart A, Tarpey P (2012). Intratumor heterogeneity and branched evolution revealed by multiregion sequencing. N Engl J Med.

[88] Yu J, Yu J, Almal AA, Dhanasekaran SM, Ghosh D, Worzel WP, Chinnaiyan AM (2007). Feature selection and molecular classification of cancer using genetic programming. Neoplasia.

[89] Lin X, Zhao Y, Song WM, Zhang B (2015). Molecular classification and prediction in gastric cancer. Comput Struct Biotechnol J.

[90] Bender RA, Erlander MG (2009). Molecular classification of unknown primary cancer. Semin Oncol.

[91] Pusztai L, Mazouni C, Anderson K, Wu Y, Symmans WF (2006). Molecular classification of breast cancer: limitations and potential. Oncologist.

[92] Viale G (2012). The current state of breast cancer classification. Ann Oncol.

[93] Brower V (2001). Proteomics: biology in the post-genomic era—companies all over the world rush to lead the way in the new post-genomics race. EMBO Rep.

[94] Medini D, Serruto D, Parkhill J, Relman DA, Donati C, Moxon R, Falkow S, Rappuoli R (2008). Microbiology in the post-genomic era. Nat Rev Microbiol.

[95] Eisenberg D, Marcotte EM, Xenarios I, Yeates TO (2000). Protein function in the post-genomic era. Nature.

[96] Kiechle FL, Zhang XB (2002). The postgenomic era—implications for the clinical laboratory. Arch Pathol Lab Med.

[97] Neet KE, Lee JC (2002). Biophysical characterization of proteins in the post-genomic era of proteomics. Mol Cell Proteomics.

[98] Vanoss CJ, Good RJ, Chaudhury MK (1987). Mechanism of DNA (Southern) and protein (Western) blotting on cellulose nitrate and other membranes. J Chromatogr.

[99] Yolken RH (1980). Enzyme-linked immunosorbent-assay (Elisa)—a practical tool for rapid diagnosis of viruses and other infectious agents. Yale J Biol Med.

[100] Su C, Fan M, Lu L, Li P (2016). Effects of silencing MTA1 gene by RNA interference on invasion and metastasis of endometrial carcinoma. Eur J Gynaecol Oncol.

[101] Nakatsura T, Senju S, Yamada K, Jotsuka T, Ogawa M, Nishimura Y (2001). Gene cloning of immunogenic antigens overexpressed in pancreatic cancer. Biochem Bioph Res Co.

[102] Deepak SA, Kottapalli KR, Rakwal R, Oros G, Rangappa KS, Iwahashi H, Masuo Y, Agrawal GK (2007). Real-time PCR: revolutionizing detection and expression analysis of genes. Curr Genomics.

[103] Kearney L, Shipley J (2012). Fluorescence in situ hybridization for cancer-related studies. Methods Mol Biol.

[104] Hermsen MA, Meijer GA, Baak JP, Joenje H, Walboomers JJ (1996). Comparative genomic hybridization: a new tool in cancer pathology. Hum Pathol.

[105] Kannan TP, Zilfalil BA (2009). Cytogenetics: past, present and future. Malays J Med Sci.

[106] Agada FO, Patmore H, Alhamarneh O, Stafford ND, Greenman J (2009). Genetic profile of head and neck squamous cell carcinoma: clinical implications. J Laryngol Otol.

[107] Poage GM, Christensen BC, Houseman EA, McClean MD, Wiencke JK, Posner MR, Clark JR, Nelson HH, Marsit CJ, Kelsey KT (2010). Genetic and epigenetic somatic alterations in head and neck squamous cell carcinomas are globally coordinated but not locally targeted. PLoS ONE.

[108] Nagai MA (1999). Genetic alterations in head and neck squamous cell carcinomas. Braz J Med Biol Res.

[109] Perez-Sayans M, Somoza-Martin JM, Barros-Angueira F, Reboiras-Lopez MD, Gandara Rey JM, Garcia-Garcia A (2009). Genetic and molecular alterations associated with oral squamous cell cancer (review). Oncol Rep.

[110] Koy S, Plaschke J, Luksch H, Friedrich K, Kuhlisch E, Eckelt U, Martinez R (2008). Microsatellite instability and loss of heterozygosity in squamous cell carcinoma of the head and neck. Head Neck.

[111] Park BJ, Chiosea SI, Grandis JR (2010). Molecular changes in the multistage pathogenesis of head and neck cancer. Cancer Biomark.

[112] Jurel SK, Gupta DS, Singh RD, Singh M, Srivastava S (2014). Genes and oral cancer. Indian J Hum Genet.

[113] Stadler ME, Patel MR, Couch ME, Hayes DN (2008). Molecular biology of head and neck cancer: risks and pathways. Hematol Oncol Clin North Am.

[114] Thomas GR, Nadiminti H, Regalado J (2005). Molecular predictors of clinical outcome in patients with head and neck squamous cell carcinoma. Int J Exp Pathol.

[115] Brill LB, Kanner WA, Fehr A, Andren Y, Moskaluk CA, Loning T, Stenman G, Frierson HF (2011). Analysis of MYB expression and MYB-NFIB gene fusions in adenoid cystic carcinoma and other salivary neoplasms. Mod Pathol.

[116] Stenman G (2013). Fusion oncogenes in salivary gland tumors: molecular and clinical consequences. Head Neck Pathol.

[117] Skalova A, Vanecek T, Sima R, Laco J, Weinreb I, Perez-Ordonez B, Starek I, Geierova M, Simpson RH, Passador-Santos F (2010). Mammary analogue secretory carcinoma of salivary glands, containing the ETV6-NTRK3 fusion gene: a hitherto undescribed salivary gland tumor entity. Am J Surg Pathol.

[118] Katabi N, Ghossein R, Ho A, Dogan S, Zhang L, Sung YS, Antonescu CR (2015). Consistent PLAG1 and HMGA2 abnormalities distinguish carcinoma ex-pleomorphic adenoma from its de novo counterparts. Hum Pathol.

[119] Matsuyama A, Hisaoka M, Nagao Y, Hashimoto H (2011). Aberrant PLAG1 expression in pleomorphic adenomas of the salivary gland: a molecular genetic and immunohistochemical study. Virchows Arch.

[120] Antonescu CR, Katabi N, Zhang L, Sung YS, Seethala RR, Jordan RC, Perez-Ordonez B, Have C, Asa SL, Leong IT (2011). EWSR1-ATF1 fusion is a novel and consistent finding in hyalinizing clear-cell carcinoma of salivary gland. Genes Chromosom Cancer.

[121] French CA (2010). NUT midline carcinoma. Cancer Genet Cytogenet.

[122] Haack H, Johnson LA, Fry CJ, Crosby K, Polakiewicz RD, Stelow EB, Hong SM, Schwartz BE, Cameron MJ, Rubin MA (2009). Diagnosis of NUT midline carcinoma using a NUT-specific monoclonal antibody. Am J Surg Pathol.

[123] Okabe M, Miyabe S, Nagatsuka H, Terada A, Hanai N, Yokoi M, Shimozato K, Eimoto T, Nakamura S, Nagai N (2006). MECT1-MAML2 fusion transcript defines a favorable subset of mucoepidermoid carcinoma. Clin Cancer Res.

[124] Kamps R, Brandao RD, Bosch BJ, Paulussen AD, Xanthoulea S, Blok MJ, Romano A (2017). Next-generation sequencing in oncology: genetic diagnosis, risk prediction and cancer classification. Int J Mol Sci.

[125] Koboldt DC, Steinberg KM, Larson DE, Wilson RK, Mardis ER (2013). The next-generation sequencing revolution and its impact on genomics. Cell.

[126] Trevino V, Falciani F, Barrera-Saldana HA (2007). DNA microarrays: a powerful genomic tool for biomedical and clinical research. Mol Med.

[127] Bumgarner R (2013). Overview of DNA microarrays: types, applications, and their future. Curr Protoc Mol Biol.

[128] LaFramboise T (2009). Single nucleotide polymorphism arrays: a decade of biological, computational and technological advances. Nucleic Acids Res.

[129] Gawad C, Koh W, Quake SR (2016). Single-cell genome sequencing: current state of the science. Nat Rev Genet.

[130] Schwartzman O, Tanay A (2015). Single-cell epigenomics: techniques and emerging applications. Nat Rev Genet.

[131] Heath JR, Ribas A, Mischel PS (2016). Single-cell analysis tools for drug discovery and development. Nat Rev Drug Discov.

[132] Pecina-Slaus N, Pecina M (2015). Only one health, and so many omics. Cancer Cell Int.

[133] Schneider MV, Orchard S (2011). Omics technologies, data and bioinformatics principles. Methods Mol Biol.

[134] Garcia I, Kuska R, Somerman MJ (2013). Expanding the foundation for personalized medicine: implications and challenges for dentistry. J Dent Res.

[135] Strausberg RL, Simpson AJ, Wooster R (2003). Sequence-based cancer genomics: progress, lessons and opportunities. Nat Rev Genet.

[136] Patel L, Parker B, Yang D, Zhang W (2013). Translational genomics in cancer research: converting profiles into personalized cancer medicine. Cancer Biol Med.

[137] Singaraju S, Prasad H, Singaraju M (2012). Evolution of dental informatics as a major research tool in oral pathology. J Oral Maxillofac Pathol.

[138] Cervigne NK, Machado J, Goswami RS, Sadikovic B, Bradley G, Perez-Ordonez B, Galloni NN, Gilbert R, Gullane P, Irish JC (2014). Recurrent genomic alterations in sequential progressive leukoplakia and oral cancer: drivers of oral tumorigenesis?. Hum Mol Genet.

[139] Mirghani H, Ugolin N, Ory C, Lefevre M, Baulande S, Hofman P, St Guily JL, Chevillard S, Lacave R (2014). A predictive transcriptomic signature of oropharyngeal cancer according to HPV16 status exclusively. Oral Oncol.

[140] Scherer A, Christensen GB (2016). Concepts and relevance of genome-wide association studies. Sci Prog.

[141] Lesseur C, Diergaarde B, Olshan AF, Wunsch-Filho V, Ness AR, Liu G, Lacko M, Eluf-Neto J, Franceschi S, Lagiou P (2016). Genome-wide association analyses identify new susceptibility loci for oral cavity and pharyngeal cancer. Nat Genet.

[142] Buermans HP, den Dunnen JT (2014). Next generation sequencing technology: advances and applications. Biochim Biophys Acta.

[143] Behjati S, Tarpey PS (2013). What is next generation sequencing?. Arch Dis Child Educ Pract Ed.

[144] Goodwin S, McPherson JD, McCombie WR (2016). Coming of age: ten years of next-generation sequencing technologies. Nat Rev Genet.

[145] Sweeney RT, McClary AC, Myers BR, Biscocho J, Neahring L, Kwei KA, Qu K, Gong X, Ng T, Jones CD (2014). Identification of recurrent SMO and BRAF mutations in ameloblastomas. Nat Genet.

[146] Gomes CC, Diniz MG, Gomez RS (2014). Progress towards personalized medicine for ameloblastoma. J Pathol.

[147] Zhang P, Mirani N, Baisre A, Fernandes H (2014). Molecular heterogeneity of head and neck squamous cell carcinoma defined by next-generation sequencing. Am J Pathol.

[148] Al-Hebshi NN, Li S, Nasher AT, El-Setouhy M, Alsanosi R, Blancato J, Loffredo C (2016). Exome sequencing of oral squamous cell carcinoma in users of Arabian snuff reveals novel candidates for driver genes. Int J Cancer.

[149] Demeure MJ, Aziz M, Rosenberg R, Gurley SD, Bussey KJ, Carpten JD (2014). Whole-genome sequencing of an aggressive BRAF wild-type papillary thyroid cancer identified EML4-ALK translocation as a therapeutic target. World J Surg.

[150] Stephens PJ, Davies HR, Mitani Y, Van Loo P, Shlien A, Tarpey PS, Papaemmanuil E, Cheverton A, Bignell GR, Butler AP (2013). Whole exome sequencing of adenoid cystic carcinoma. J Clin Invest.

[151] Nichols AC, Chan-Seng-Yue M, Yoo J, Xu W, Dhaliwal S, Basmaji J, Szeto CC, Dowthwaite S, Todorovic B, Starmans MH (2012). A pilot study comparing HPV-positive and HPV-negative head and neck squamous cell carcinomas by whole exome sequencing. ISRN Oncol.

[152] Fingerman IM, Zhang X, Ratzat W, Husain N, Cohen RF, Schuler GD (2013). NCBI epigenomics: what’s new for 2013. Nucleic Acids Res.

[153] Park YJ, Claus R, Weichenhan D, Plass C (2011). Genome-wide epigenetic modifications in cancer. Prog Drug Res.

[154] Adeyemo WL, Butali A (2017). Genetics and genomics etiology of nonsyndromic orofacial clefts. Mol Genet Genomic Med.

[155] Sandoval J, Esteller M (2012). Cancer epigenomics: beyond genomics. Curr Opin Genet Dev.

[156] Kozaki K, Imoto I, Mogi S, Omura K, Inazawa J (2008). Exploration of tumor-suppressive microRNAs silenced by DNA hypermethylation in oral cancer. Cancer Res.

[157] Mascolo M, Siano M, Ilardi G, Russo D, Merolla F, De Rosa G, Staibano S (2012). Epigenetic disregulation in oral cancer. Int J Mol Sci.

[158] Singh NN, Peer A, Nair S, Chaturvedi RK (2016). Epigenetics: a possible answer to the undeciphered etiopathogenesis and behavior of oral lesions. J Oral Maxillofac Pathol.

[159] Gasche JA, Goel A (2012). Epigenetic mechanisms in oral carcinogenesis. Future Oncol.

[160] Shaw R (2006). The epigenetics of oral cancer. Int J Oral Maxillofac Surg.

[161] Lindsay C, Seikaly H, Biron VL (2017). Epigenetics of oropharyngeal squamous cell carcinoma: opportunities for novel chemotherapeutic targets. J Otolaryngol Head Neck Surg.

[162] Duncan MJ (2003). Genomics of oral bacteria. Crit Rev Oral Biol Med.

[163] Kapatral V, Anderson I, Ivanova N, Reznik G, Los T, Lykidis A, Bhattacharyya A, Bartman A, Gardner W, Grechkin G (2002). Genome sequence and analysis of the oral bacterium Fusobacterium nucleatum strain ATCC 25586. J Bacteriol.

[164] Duran-Pinedo AE, Chen T, Teles R, Starr JR, Wang X, Krishnan K, Frias-Lopez J (2014). Community-wide transcriptome of the oral microbiome in subjects with and without periodontitis. ISME J.

[165] Kerr AR (2015). The oral microbiome and cancer. J Dent Hyg.

[166] Schmidt BL, Kuczynski J, Bhattacharya A, Huey B, Corby PM, Queiroz EL, Nightingale K, Kerr AR, DeLacure MD, Veeramachaneni R (2014). Changes in abundance of oral microbiota associated with oral cancer. PLoS ONE.

[167] Chi LM, Lee CW, Chang KP, Hao SP, Lee HM, Liang Y, Hsueh C, Yu CJ, Lee IN, Chang YJ (2009). Enhanced interferon signaling pathway in oral cancer revealed by quantitative proteome analysis of microdissected specimens using 16O/18O labeling and integrated two-dimensional LC-ESI-MALDI tandem MS. Mol Cell Proteomics.

[168] Balog J, Sasi-Szabo L, Kinross J, Lewis MR, Muirhead LJ, Veselkov K, Mirnezami R, Dezso B, Damjanovich L, Darzi A (2013). Intraoperative tissue identification using rapid evaporative ionization mass spectrometry. Sci Transl Med.

[169] Tiziani S, Lopes V, Gunther UL (2009). Early stage diagnosis of oral cancer using 1H NMR-based metabolomics. Neoplasia.

[170] Amann A, Miekisch W, Schubert J, Buszewski B, Ligor T, Jezierski T, Pleil J, Risby T (2014). Analysis of exhaled breath for disease detection. Annu Rev Anal Chem.

[171] Krilaviciute A, Heiss JA, Leja M, Kupcinskas J, Haick H, Brenner H (2015). Detection of cancer through exhaled breath: a systematic review. Oncotarget.

[172] van Mastrigt E, Reyes-Reyes A, Brand K, Bhattacharya N, Urbach HP, Stubbs AP, de Jongste JC, Pijnenburg MW (2016). Exhaled breath profiling using broadband quantum cascade laser-based spectroscopy in healthy children and children with asthma and cystic fibrosis. J Breath Res.

[173] Zdrojewicz Z, Waracki M, Bugaj B, Pypno D, Cabala K (2015). Medical applications of nanotechnology. Postepy Hig Med Dosw.

[174] Emerich DF, Thanos CG (2003). Nanotechnology and medicine. Expert Opin Biol Ther.

[175] Saini R, Saini S, Sharma S (2010). Nanotechnology: the future medicine. J Cutan Aesthet Surg.

[176] Patil M, Mehta DS, Guvva S (2008). Future impact of nanotechnology on medicine and dentistry. J Indian Soc Periodontol.

[177] Virupakshappa B (2012). Applications of nanomedicine in oral cancer. Oral Health Dent Manag.

[178] Kah JC, Kho KW, Lee CG, James C, Sheppard R, Shen ZX, Soo KC, Olivo MC (2007). Early diagnosis of oral cancer based on the surface plasmon resonance of gold nanoparticles. Int J Nanomedicine.

[179] Su X, Zhang J, Sun L, Koo TW, Chan S, Sundararajan N, Yamakawa M, Berlin AA (2005). Composite organic-inorganic nanoparticles (COINs) with chemically encoded optical signatures. Nano Lett.

[180] Virlan MJ, Miricescu D, Radulescu R, Sabliov CM, Totan A, Calenic B, Greabu M: Organic Nanomaterials and Their Applications in the Treatment of Oral Diseases. Molecules 2016, 21(2). doi:10.3390/molecules21020207. 10.3390/molecules21020207 PMC6273611 26867191

[181] Park W, Owens JM (2006). Future directions in the treatment of oral cancer. Otolaryngol Clin North Am.

[182] Christian K, Johanna M, Werner A, Kathrin B, Tesfay GM, Robert H, Abbas A, Stefan W, Andreas B, Wilhelm NF (2014). Raman difference spectroscopy: a non-invasive method for identification of oral squamous cell carcinoma. Biomed Opt Express.

[183] Kircher MF, Hricak H, Larson SM (2012). Molecular imaging for personalized cancer care. Mol Oncol.

[184] Awan KH, Morgan PR, Warnakulasuriya S (2011). Evaluation of an autofluorescence based imaging system (VELscope) in the detection of oral potentially malignant disorders and benign keratoses. Oral Oncol.

[185] Drezek R, Sokolov K, Utzinger U, Boiko I, Malpica A, Follen M, Richards-Kortum R (2001). Understanding the contributions of NADH and collagen to cervical tissue fluorescence spectra: modeling, measurements, and implications. J Biomed Opt.

[186] Huber MA (2012). Adjunctive diagnostic aids in oral cancer screening: an update. Tex Dent J.

[187] Rosenkrantz AB, Friedman K, Chandarana H, Melsaether A, Moy L, Ding YS, Jhaveri K, Beltran L, Jain R (2016). Current status of hybrid PET/MRI in oncologic imaging. AJR Am J Roentgenol.

[188] Almuhaideb A, Papathanasiou N, Bomanji J (2011). 18F-FDG PET/CT imaging in oncology. Ann Saudi Med.

[189] Czernin J, Phelps ME (2002). Positron emission tomography scanning: current and future applications. Annu Rev Med.

[190] Czernin J (2002). Clinical applications of FDG-PET in oncology. Acta Med Austriaca.

[191] Gambhir SS (2002). Molecular imaging of cancer with positron emission tomography. Nat Rev Cancer.

[192] Rohren EM, Turkington TG, Coleman RE (2004). Clinical applications of PET in oncology. Radiology.

[193] Arens AI, Troost EG, Schinagl D, Kaanders JH, Oyen WJ (2011). FDG-PET/CT in radiation treatment planning of head and neck squamous cell carcinoma. Q J Nucl Med Mol Imaging.

[194] Rajpura KB, Patel PS, Chawda JG, Shah RM (2005). Clinical significance of total and lipid bound sialic acid levels in oral pre-cancerous conditions and oral cancer. J Oral Pathol Med.

[195] Wilma Delphine Silvia CR, Vasudevan DM, Prabhu KS (2001). Evaluation of serum glycoproteins in oral carcinoma. Indian J Clin Biochem.

[196] Joshi M, Patil R (2010). Estimation and comparative study of serum total sialic acid levels as tumor markers in oral cancer and precancer. J Cancer Res Ther.

[197] Kaneko OF, Willmann JK (2012). Ultrasound for molecular imaging and therapy in cancer. Quant Imaging Med Surg.

[198] European Society of R (2015). Medical imaging in personalised medicine: a white paper of the research committee of the European Society of Radiology (ESR). Insights Imaging.

[199] Baeten J, Suresh A, Johnson A, Patel K, Kuriakose M, Flynn A, Kademani D (2014). Molecular imaging of oral premalignant and malignant lesions using fluorescently labeled lectins. Transl Oncol.

[200] Van Audenhove I, Gettemans J (2016). Nanobodies as versatile tools to understand, diagnose, visualize treat cancer. EBioMedicine.

[201] Bhagat AA, Lim CT (2012). Microfluidic technologies. Recent Results Cancer Res.

[202] Robinson T, Dittrich PS (2013). Microfluidic technology for molecular diagnostics. Adv Biochem Eng Biotechnol.

[203] Luka G, Ahmadi A, Najjaran H, Alocilja E, DeRosa M, Wolthers K, Malki A, Aziz H, Althani A, Hoorfar M (2015). Microfluidics integrated biosensors: a leading technology towards lab-on-a-chip and sensing applications. Sensors.

[204] Ji S, Choi Y (2015). Point-of-care diagnosis of periodontitis using saliva: technically feasible but still a challenge. Front Cell Infect Microbiol.

[205] Huddy JR, Ni MZ, Markar SR, Hanna GB (2015). Point-of-care testing in the diagnosis of gastrointestinal cancers: current technology and future directions. World J Gastroenterol.

[206] Rusling JF, Kumar CV, Gutkind JS, Patel V (2010). Measurement of biomarker proteins for point-of-care early detection and monitoring of cancer. Analyst.

[207] Otsu K, Kumakami-Sakano M, Fujiwara N, Kikuchi K, Keller L, Lesot H, Harada H (2014). Stem cell sources for tooth regeneration: current status and future prospects. Front Physiol.

[208] Prince ME, Sivanandan R, Kaczorowski A, Wolf GT, Kaplan MJ, Dalerba P, Weissman IL, Clarke MF, Ailles LE (2007). Identification of a subpopulation of cells with cancer stem cell properties in head and neck squamous cell carcinoma. Proc Natl Acad Sci USA.

[209] Mery B, Guy JB, Espenel S, Wozny AS, Simonet S, Vallard A, Alphonse G, Ardail D, Rodriguez-Lafrasse C, Magne N (2016). Targeting head and neck tumoral stem cells: from biological aspects to therapeutic perspectives. World J Stem Cells.

[210] Trixler F (2013). Quantum tunnelling to the origin and evolution of life. Curr Org Chem.

[211] Cooper WG (1993). Roles of evolution, quantum mechanics and point mutations in origins of cancer. Cancer Biochem Biophys.

[212] Davies P, Demetrius LA, Tuszynski JA (2012). Implications of quantum metabolism and natural selection for the origin of cancer cells and tumor progression. AIP Adv.

[213] Barrett T, Edgar R (2006). Mining microarray data at NCBI’s gene expression omnibus (GEO)*. Methods Mol Biol.

[214] Sayers EW, Barrett T, Benson DA, Bryant SH, Canese K, Chetvernin V, Church DM, DiCuccio M, Edgar R, Federhen S (2009). Database resources of the National Center for Biotechnology Information. Nucleic Acids Res.

[215] Lee H, Palm J, Grimes SM, Ji HP (2015). The Cancer Genome Atlas Clinical Explorer: a web and mobile interface for identifying clinical-genomic driver associations. Genome Med.

[216] Knaul FM, Atun R, Farmer P, Frenk J (2013). Seizing the opportunity to close the cancer divide. Lancet.

[217] Haspel RL, Rinder HM, Frank KM, Wagner J, Ali AM, Fisher PB, Parks ER, Training Residents in Genomics Working G (2014). The current state of resident training in genomic pathology: a comprehensive analysis using the resident in-service examination. Am J Clin Pathol.

[218] Haspel RL (2013). Teaching residents genomic pathology: a novel approach for new technology. Adv Anat Pathol.

[219] Mardis ER (2006). Anticipating the 1,000 dollar genome. Genome Biol.

[220] Robertson JA (2003). The $1000 genome: ethical and legal issues in whole genome sequencing of individuals. Am J Bioeth.

